# The Significance of Sleep Disorders
*A Paper read at a Meeting of the Bristol Medico-Chirurgical Society held at the University of Bristol on 10th April, 1929.


**Published:** 1929

**Authors:** Hugh H. Carleton

**Affiliations:** Assistant Physician, Bristol General Hospital


### The Bristol Medico-Chirurgical Journal

*"Scire est nescire, nisi id me
Scire alius sciret."*

AUTUMN, 1929.

### THE SIGNIFICANCE OF SLEEP DISORDERS.*

BY

Hugh H. Carleton, M.D., M.R.C.P.,
*Assistant Physician, Bristol General Hospital.*

Thr purpose of this paper is an attempt to discover whether there are any distinctions whereby the various factors in sleep and insomnia may be distinguished; and if such distinctions can be established, to consider their bearing on treatment.

Whatever views we may hold about sleep, we shall probably all agree that it is signalized by a transient cessation of consciousness, an essentially cortical phenomenon; but we have to consider how far normal sleep is purely cortical and how far it is also related to changes at subcortical levels.

There is some reason for the belief that certain regions of the brain may be called subcortical sleep

* A Paper read at a Meeting of the Bristol Medico-Chirurgical Society held at the University of Bristol on 10th April, 1929.

centres. These sleep centres are situated on pathways afferent from the muscles and vegetative nervous system to the regions of the brain situated around the third ventricle and hypothalamus, and thence relayed to the highest neurological levels in the cerebral cortex. The tendency to sleep is facilitated, or the reverse, by this incoming stream of messages or excitations, from the vegetative nervous system, mediated through the hypothalamus.

It appears necessary to postulate the existence of neural tone as an essential factor in the facilitation or prevention of sleep. Our own subjective sensations harmonize with such a view. Temperamental differences suggest, not only that neural tone exists, but that it is set at varying levels in different individuals and in the same individuals at different times. It is intimately related to physical states, as determined by muscle sense, and the effect of endocrine influence on bodily functions, also on the balance struck by the regulating activities of the vegetative nervous system.

Temperamental differences express themselves in the varying degrees of response manifested by different people. The speed and strength of irradiation of impulses through the association fibres of the cerebral cortex is, so to speak, a touchstone for estimating nervous tone or temperament.

The state of neural tone determines our feeling of well-being, and is itself mainly determined by muscle and visceral sensations and the inherent quality of our brain stuff. When our internal machinery is running smoothly, beyond a general feeling of well-being we are unconscious of our bodily sensations. On the contrary, in the presence of disease affecting viscera, of endocrine disturbance, or in the face of muscular discomfort, the feeling of well-being is upset. A stream of noxious impulses arises, which reaches, in the first place, the subcortical vegetative centres. This unfavourable stimulation of subcortical centres causes an upset of neural tone, and prevents the development of inhibitory states in the cortex, which normally result in sleep.

Normal sleep can be accounted for by cortical changes alone, provided neural tone remains constant for the time being, that is to say, provided no profound disturbances in the muscular and vegetative fields arise.

It is not easy to give a completely satisfactory definition of normal sleep, but for practical purposes it may be described as "a temporary cessation of consciousness occurring as a result of fatigue, monotony or habit."

The study of sleep under exact experimental conditions by Pavlov,[1] in his work on conditioned reflexes, has thrown considerable light on the fundamental processes underlying normal sleep. Pavlov's researches may be summarized thus: He set out to make a purely objective study of the activities of the cerebral cortex. Working with dogs, the method was to establish a large variety of conditioned reflexes, *i.e.* to obtain measured responses to accurately-controlled stimuli or signals. For the establishment of conditioned reflexes an intact cerebral cortex is essential. In the earlier stages of training precise, delicately-adjusted responses were readily obtained. When the duration of the experiments was prolonged, so that the element of fatigue was introduced, or the conditions savoured of monotony, or on the presentation of signals habitually associated with tiredness or boredom, the animals quickly passed into a state of internal inhibition or refractoriness, indistinguishable from sleep. Pavlov came to the conclusion that this state of internal inhibition was a purely cortical phenomenon, and was identical with normal sleep.

By the phrase "internal inhibition" Pavlov obviously means a negative state or cessation of neural activity; in other words, a resting phase. This use of the term inhibition needs to be distinguished from the function of inhibitory control, an important physiological principle, underlying the evolutionary development of the nervous system, the latter being regarded as built up of superimposed levels, each higher level exerting inhibitory control over levels of lower physiological grade.

Let us consider internal inhibition, and see if the seat of the inhibitory state can be more closely defined. A brief survey of the structure of the cerebral cortex will be helpful. Schematically, we may think of the cortex as composed of arches, each consisting of an afferent and an efferent column, the interval between the two being spanned by association neurones, in the main made up of enormous numbers of closely-packed granular cells, which, with their axones, are confined to the grey matter and contribute to the laminated appearance seen in sections of the cortex.

Speaking generally, and omitting detail, one may distinguish two types of cortex, first motor, and secondly receptive cortex. Function and structure are closely correlated. The motor cortex is characterized by a wealth of pyramidal cells, and the sensory or receptive cortex by a concentration of closely-packed granular cells.[2] There is general agreement that the granular receptive cortex is concerned with the analysis of sense impressions, that it has the power to store and liberate nervous energy in accordance with physiological need, and is, in fact, the physiological basis of mind, memory, and consciousness. With the active phase of the granular cortex we associate the attributes of consciousness, and with the resting phase the cessation of consciousness or sleep.

Association between various areas of the cortex is established on the efferent side by axones, which have their cell stations in the small pyramidal cells of the third layer of the cortex; but these cells are only activated by impulses from the granular layer, and it is the latter which determines activity or inhibition in the cortex as a whole.

The detailed study of the behaviour of this felt-work of association neurones formed a large part of Pavlov's research. It is essentially in relation to the organs of special sense that sensory[3] cortical areas are developed, and the arrangement within the cortex is such that we may speak of visual, auditory, olfactory, and tactile analysers. Experimental results show that these cortical analysers become blocked and display the phenomenon of internal inhibition as a result of fatigue, monotony or habit, and if this process of inhibition becomes widely spread sleep ensues.

This, briefly, is a description of normal sleep. In it no account is taken of the state of neural tone, already referred to, which is largely determined by the stream of impulses afferent from muscles and the vegetative nervous sytem to certain subcortical centres. The relation of these subcortical regions to the process of sleep must now be considered. It must be remembered, however, that while it may be convenient to speak of subcortical sleep centres, the latter act ultimately through the medium of the cerebral cortex, since sleep, without loss of consciousness, is inconceivable.

Experimental and clinical data indicate that sleep centres are situated at the base of the brain, in those regions which form the boundaries of the third ventricle, more particularly its floor and posterior wall, also, notably, in and around the region of the hypothalamus, parts of the brain which are to be regarded physiologically as the head end of the vegetative nervous system, and the head station for proprioceptive impressions from muscles. Clinical evidence as to the existence of subcortical sleep centres comes from three main sources :—

(1) Cerebral tumours, situated in and around the third ventricle and verified post-mortem : cases in which, during life, pathological somnolence was a prominent symptom.

(2) Observations on the narcolepsies, notably by Kinnier Wilson [4] and Adie. [5]

(3) Observations on encephalitis lethargica.

The observations on tumours in the region of the third ventricle are to be found in the reports of collected cases of Cushing, Righetti[6] and of Purves Stewart,[7] and are convincing.

For the detailed clinical evidence afforded by the narcolepsies the writings of Wilson[4] and Adie[5] in *Brain* should be consulted. In narcolepsy, side by side with the phenomenon of pathological sleep, there occurs a concurrent syndrome, cataplexy, in which the patient, under the influence of emotion, exhibits complete loss of postural tone. He may, for instance, on the exhibition of strong emotion, laughter or anger, fall to the ground all of a heap. Cataplexy is a typical third ventricular syndrome, and, as we have seen from the above, is also associated with pathological sleep. It supports the view that sleep centres are situated in this region of the brain. The post-encephalitic state is well known. In this condition we have a third ventricle syndrome, namely, altered postural tone, which, together with disorder of sleep, points to the existence of sleep-regulating centres in and around the third ventricle.

Experimental evidence as to the existence of subcortical sleep centres comes from two main sources, the work of Dubois[8] as far back as 1896, and more recently the work of Cushing and Goetsche.[9] The work consists of experimental ablations in the region of the hypophysis cerebri and third ventricle. These observers were led to the conclusion that the third ventricular region of the brain plays a predominant role in the mechanism of sleep. Further, there is no doubt as to the close relationship of this region of the brain to the vegetative nervous system.

We are forced, then, to the following general conclusions, namely, that disordered muscular sensation, visceral disturbances, functional or organic, painful visceral disease, *e.g.* angina or peritonitis, or endocrine dysfunction, *e.g.* exophthalmic goitre, may act as excitants of the vegetative subcortical sleep centres. The latter, in turn, transmit a noxious, afferent stream of impulses to the cortex, which disturbs neural tone. In consequence the normal development of internal inhibition in response to fatigue, monotony or habit is prevented, and insomnia results.

We now come to the clinical application of the above views. In the treatment of patients can we recognize disorders of sleep which correspond to types based on the foregoing considerations ? In the first place, can we recognize minor disorders of sleep, beginning and ending with disturbances at the cortical level ? Secondly, do the more profound forms of insomnia indicate disorders of muscle sense or disturbance of the vegetative nervous system, which act first on subcortical sleep centres and through them secondarily on the cerebral cortex ?

Assuming that we find such clinical evidence, what implications follow with regard to treatment ? We are all familiar with delayed or disturbed sleep, which may follow some unusual excitement, and the inability to sleep in unusual surroundings. Such minor disturbances are not associated with any physical discomfort or ill-health, and almost any of the milder sedatives are sufficient to ensure sleep. It is a question of promoting the normal tendency of the cortex to develop inhibition. Likewise, we are all familiar with states associated with disordered visceral or endocrine function, cases which are frequently termed anxiety neuroses. Patients belonging to this category frequently complain of obstinate insomnia ; their rest at night is upset by palpitation, indigestion, and general disturbances of their sense of physical well-being. Usually insomnia of this type may be corrected by moderate doses of the milder hypnotics and other general medical treatment.

A drug in common use, the action of which is interesting and merits consideration, is aspirin. Given at night, it frequently acts as a sedative and mild hypnotic ; yet many people take aspirin in the daytime without finding the slightest tendency to somnolence, such as may, for instance, be produced by the bromides. The explanation probably lies in the fact that aspirin has a selective effect in diminishing muscular sensibility. In general *malaise* and muscular rheumatism there is a greatly heightened sense of muscular discomfort, which means that a stream of noxious impulses pours in to the hypothalamus by proprioceptive pathways. The result is subcortical interference with sleep. Aspirin, by diminishing this stream, allows normal cortical inhibition to proceed, without itself acting directly at cortical levels. Such a view would explain the inefficacy of aspirin to cause somnolence when taken in the daytime.

When we pass to a consideration of serious mental disorders the problem becomes more difficult. Intractable insomnia ushers in the periodic breakdowns of manic-depressive psychosis in the majority of cases. Apart from the patients in this class, who become frankly insane, there is a still larger number who suffer from severe periodic depression, and the latter is often associated with disorder of physical sensations. In the frankly psychotic such disordered sensation often forms the basis of somatic delusions. Anyone who sees a large number of cases of manic-depressive psychosis must be impressed with the view that the condition has a definite organic basis of physical ill-health, and treatment, to be successful, must be directed towards the correction of the latter.

Psychotherapy appears to be a pure waste of time with these patients, whereas treatment directed towards improvement of their physical condition, whether it be the elimination of sepsis, restoration of endocrine balance or the cure of visceral disease, is not infrequently followed by a marked improvement in the mental state. Since severe insomnia usually ushers in the attack, the symptom calls urgently for treatment at an early stage of the breakdown. It will be found in many cases that the heaviest doses of bromide, chloral, and one of the veronal derivatives are required in order to produce sleep. By such methods some improvement is occasionally obtained. On the other hand, more frequently, after two or three weeks'

artificially-induced sleep, with heavy doses of drugs, the condition of the patient appears in no way improved. Somatic delusions and feelings of general physical and mental distress remain unchanged.

This failure of treatment may be accounted for to some extent if we remember that the drugs mentioned — bromides, chloral, veronal, and the like — act essentially at the cortical level, whereas in the type of case under consideration the primary disturbance of health would appear to be deep-seated, and to act through the vegetative nervous system on subcortical centres. Are there any drugs at our disposal which are more likely to meet the requirements, which, in fact, will produce their sedative effect at these lower levels ? Obviously there are such drugs, notably opium and hyoscine taken by the mouth, and belladonna. In the writer's experience these drugs have certainly proved more useful than any others in the treatment of manic-depressive psychosis, particularly if used in conjunction with small doses of bromide. The use of opium by mouth in the form of Dover's powder is free from objection in the treatment of insomnia in such cases, and the same applies to hyoscine, the latter notably in the post-encephalitic state, which is so frequently associated with great muscular discomfort. The results of the diseases in question are infinitely more disastrous to the patient than the very slight risk of inducing an opium or hyoscine habit. Belladonna has had a reputation in the treatment of manic-depressive psychoses for many years, though the reason for its utility has probably not been fully appreciated.

Without doubt endocrine disturbances promote a number of the psychoses. In spite of our increased knowledge of endocrine balance, we are still faced with considerable difficulty in the efficient administration of glandular extracts. In a number of the emotional states associated with mental disorder we are able to surmise the probable over-action of the thyroid and suprarenals, which furnish two important katabolic hormones. Have we any means of counteracting this over-activity ?

Experience in recent times indicates that perhaps our chief anabolic hormone is insulin. There is evidence that insulin is of real service in treatment, particularly the pre- and post-operative treatment of Graves's disease. It is also the physiological antidote to adrenalin. It is put forward as a suggestion that the administration of glucose and insulin may find a useful place in the treatment of mania and other states of grave mental agitation and anxiety. To sum up the problem of insomnia in manic-depressive psychoses : treatment, to be effective, must aim at the correction of any organic physical disturbance of the patient's vegetative life. If material improvement is to be achieved, drugs which act at the vegetative level play a more important part than those which act solely on the cerebral cortex.

A few words must be said on the rationale of treatment of painful medical and surgical diseases in which sleep is prevented by reason of physical pain. It is a matter of common knowledge that occasional acute mental breakdown ushered in by insomnia follows major abdominal operations, and that mental disorder is not infrequently associated with the graver forms of heart disease. There can be little doubt that opium, or its allies, should be the sheet-anchor in most of these cases.

In conclusion, when we are called upon to treat insomnia, it is important to consider carefully whether the symptom is simply the result of some minor disturbance of the three factors, fatigue, monotony or habit, which determine the development of normal sleep, in which case cortical levels alone are involved ; or whether there is a more profound disturbance arising in the fields of the vegetative nervous system which, in the first place, acts on the subcortical sleep centres, and only secondarily on the highest cortical levels.

### BIBLIOGRAPHY.

[1] Pavlov, *Conditioned Reflexes*, Oxford University Press, 1927.

[2] Berry, *Brain and Mind*, Macmillan, 1928.

[3] von Economo, *The Cytoarchitectonics of the Human Cerebral Cortex*, Oxford University Press, 1929.

[4] Kinnier Wilson, " The Narcolepsies," *Brain*, 1928, li. 63.

[5] Adie, " Idiopathic Narcolepsy," *Brain*, 1926, p. 257.

[6] Righetti, " Contributo clinico e anatoma-patologico allo studio dei gliomi cerebrali," *Riv. Pat. Neur. e Ment.*, 1903.

[7] Purves Stewart, *Intracranial Tumours*, Oxford University Press, 1927.

[8] Dubois, " Étude sur le mécanisme de la thermogénèse et du sommeil chez les mammifère," *Ann. Univ. de Lyon*, 1896, p. 175.

[9] Cushing and Goetsche, " Hibernation and the Pituitary Body," *Journ. Exper. Med.*, 1915, xxii. 25.

